# An analysis of the correlation between diabetic retinopathy and preretinal oxygen tension using three-dimensional spoiled gradient-recalled echo sequence imaging

**DOI:** 10.1186/s12880-022-00846-x

**Published:** 2022-07-05

**Authors:** Min-Jie Zhou, Ju-Wei Shao, Jian Pu, Shu-Tian Xiang, Yi Liang, Qian He, Wei Su, Cheng Liu

**Affiliations:** Department of Radiology, Affiliated Hospital of Yunnan University, The Second People’s Hospital of Yunnan Province, No.176 of Qingnian street, Wuhua District, Kunming, 650000 China

**Keywords:** Hyperglycemia, Diabetic retinopathy, Oxygen tension, Magnetic resonance imaging, Three-dimensional spoiled gradient-recalled echo

## Abstract

**Background:**

The aims of this study were to evaluate the levels of preretinal oxygen tension in patients with diabetes who did not have hypertension by using three-dimensional spoiled gradient-recalled (3D-SPGR) echo sequence imaging and to explore the correlation between diabetic retinopathy (DR) and changes in preretinal oxygen tension.

**Method:**

This study involved 15 patients with type 2 diabetes without hypertension, who were divided into a diabetic retinopathy (DR) group (*n* = 10 eyes) and a diabetic non-retinopathy (NDR) group (*n* = 20 eyes), according to the results of a fundus photography test. Another healthy control group (*n* = 14 eyes) also participated in the study. The preretinal vitreous optic disc area, nasal side, and temporal side signal intensity of the eyes was assessed before and after oxygen inhalation with the use of 3D-SPGR echo magnetic resonance imaging (MRI). The signal acquisition time was 10, 20, 30, 40, and 50 min after oxygen inhalation.

**Results:**

The results showed that, in the DR and NDR groups, the preretinal vitreous oxygen tension increased rapidly at 10 min after oxygen inhalation and peaked at 30–40 min, and the increased slope of the DR group was higher than that of the NDR group. The oxygen tension of the preretinal vitreous gradually increased after oxygen inhalation, and the difference between the DR and NDR groups and the control group was statistically significant (*P* < 0.05). The preretinal vitreous oxygen tension was higher in the optic disc, temporal side, and nasal side in the NDR group than in the control group, and the difference was statistically significant (*P* < 0.05). The maximum slope ratios of the optic disc and the temporal side of the DR group were greater than those of the control group, and the difference was statistically significant (*P* < 0.05).

**Conclusion:**

Three-dimensional-SPGR echo MRI sequencing technology is useful for detecting preretinal oxygen tension levels in patients with diabetes. It can be used as one of the functional and imaging observation indicators for the early diagnosis of DR.

## Background

Diabetic retinopathy (DR) refers to retinal microvascular damage caused by diabetes, which can lead to visual impairment and blindness. According to International Diabetes Federation (IDF) estimates, the global diabetes population will rise from 463 million in 2019 to 700 million by 2045, and the global number of adults with diabetic retinopathy (DR), which is the leading cause of blindness in the working age group, will reach 160 million [1, 2]. However, early detection and appropriate intervention can significantly reduce severe vision loss and prevent more than 90% of cases of blindness caused by DR [3, 4].

Diabetic retinopathy occurs when hyperglycemia leads to retinal capillary damage, microcirculation disturbance, and retinal vascular hemodynamic changes [5]. As a key nutrient, oxygen is extremely important for the retina, which is one of the most metabolized tissues in the human body [6, 7]. Studies have shown that changes in retinal oxygen saturation may precede the progression of diabetic retinopathy and that oxygen saturation is more sensitive to disease progression than retinopathy grading [8]. Retinal ischemia and hypoxia can be assessed by measuring the intraretinal oxygenation response (△PO_2_).

At present, fundus photography (FA) and fundus fluorescein angiography (FFA) are the main detection methods for the diagnosis of diabetic retinopathy—but due to their invasiveness, ocular media (cataract, silicone oil eye or vitreous hemorrhage) limitations and the inability to perform functional and morphological analysis [9], which cannot meet the requirements of early diagnosis of DR or long-term monitoring of the condition. Due to the exchange of oxygen between blood and retinal tissue under normal physiological conditions, measuring the oxygen tensor in the preretinal vitreous can reflect the functional state of the retina. At present, using a fiber optic oxygen probe is considered to be the best way of measuring oxygen tension, but this method is limited because it can only be used during curative surgery, and it is invasive. With the development of MRI technology, the non-invasive detection of retinal oxygen tension changes in vivo has become a reality, and satisfactory results have been obtained. The ischemic and hypoxic state of the retina can be investigated by measuring the changes in retinal oxygen tension levels indicated by △PO_2_. Among the various methods for measuring retinal oxygen tension, MRI is a non-invasive and accurate measurement method, and its results are as reliable as using an oxygen probe electrode [10–12]. MRI detection of △PO_2_ uses paramagnetic oxygen molecules to directly detect the oxygen content in the vitreous adjacent to the retina, and it does not use blood vessels. Changes in different parts of the retina can be monitored, and so this technique can be used to evaluate diabetic retinopathy [13, 14].

Recent research on DR focused on patients with diabetes who also had hypertension, while oxygen and oxygen tension were designated research interference factors.When combined with diabetes, hypertension induces increased expression of vascular endothelial growth factor (VEGF), which can lead to hardening of retinal blood vessels and affect retinal oxygen tensor. For this reason, researchers have typically focused on cases where diabetes is combined with hypertension. However, in clinical practice, nearly half of patients with diabetes do not present with hypertension. Oxygenation in the retinas of patients with diabetes without hypertension has not been researched to date, and the value of three-dimensional spoiled gradient-recalled (3D-SPGR) echo sequence imaging for evaluating the level of △PO_2_ is as yet unknown. Accordingly, the current study was conducted to evaluate the level of △PO_2_ in patients with diabetes without hypertension using the 3D-SPGR echo sequence imaging technique to explore the correlation between DR and changes in preretinal oxygen tension. The initial hypothesis of this study was that a close correlation between retinopathy and preretinal oxygen tension would be observed among patients with diabetes without hypertension.

## Materials and methods

### Subjects

Between December 2018 and December 2019, patients with type 2 diabetes without hypertension at the Department of Endocrinology and Ophthalmology of our hospital were recruited for this study. The study group was selected according to the International Diabetic Retinopathy Disease Severity Scale [15]. This study was conducted in accordance with the Declaration of Helsinki and approved by the Ethics Committee of the hospital. All the participants provided signed informed consent for their inclusion in the study.

### Inclusion and exclusion criteria

The inclusion criteria for the study were as follows: (1) patients who were diagnosed with type 2 diabetes; (2) patients older than 18 years of ages; and (3) patients who provided signed informed consent.

The exclusion criteria for the study were as follows: (1) patients who suffered from hypertension; (2) patients who had a severe infection or patients who had severe liver or kidney dysfunction; (3) patients with previous eye diseases, such as strabismus, glaucoma, optic neuropathy, macular degeneration, or eye tumors; (4) patients with previous eye treatments such as vitreous drug injection, eye laser treatment, or surgery; and (5) patients with incomplete medical data.

The inclusion criteria for the control group were as follows: (1) those who had no abnormalities detected during ophthalmic and fundus examinations; (2) those with no family history of diabetes or hypertension; and (3) those who were not significantly different in age or gender to the patients with diabetes participating in the study.

### Magnetic resonance imaging

The MRI tests were conducted using US General Motors 1.5 Tesla dual-gradient MR scanners (GE Sigma TwinSpeed 1.5 T, GE Healthcare, Milwaukee, WI, USA), which were capable of performing high-resolution head and neck imaging, combined with an 8-channel coil using 3D T1-weighted image (T1WI) spoiler gradient echoes. For each patient, first, the MRI scan was performed three times in the normal atmosphere. Then, using a 40 L oxygen bag, medical oxygen (oxygen concentration ≥ 99.5%) was continuously inhaled through a nasal catheter at a flow rate of 0.5 L/min. Five scans were performed in quick succession every 10 min after the oxygen inhalation. During the interval between each set of scans, subjects could blink. During the scan, the image acquisition was always aimed at the cross-sectional plane of the central optic disc of both eyes. The scanning parameters were as follows: repetition time = 10.5 ms; echo time = 2.1 ms; flip angle = 15°; field of view = 20 × 20 cm; layer thickness = 5 mm, layer spacing = 2.5 mm; matrix = 256 × 256; number of excitations = 1; and single scan time = 29 s. The overall scan time per group of scans was 1 min 28 s.

### Post-processing and data analysis

**Fig. 1:**
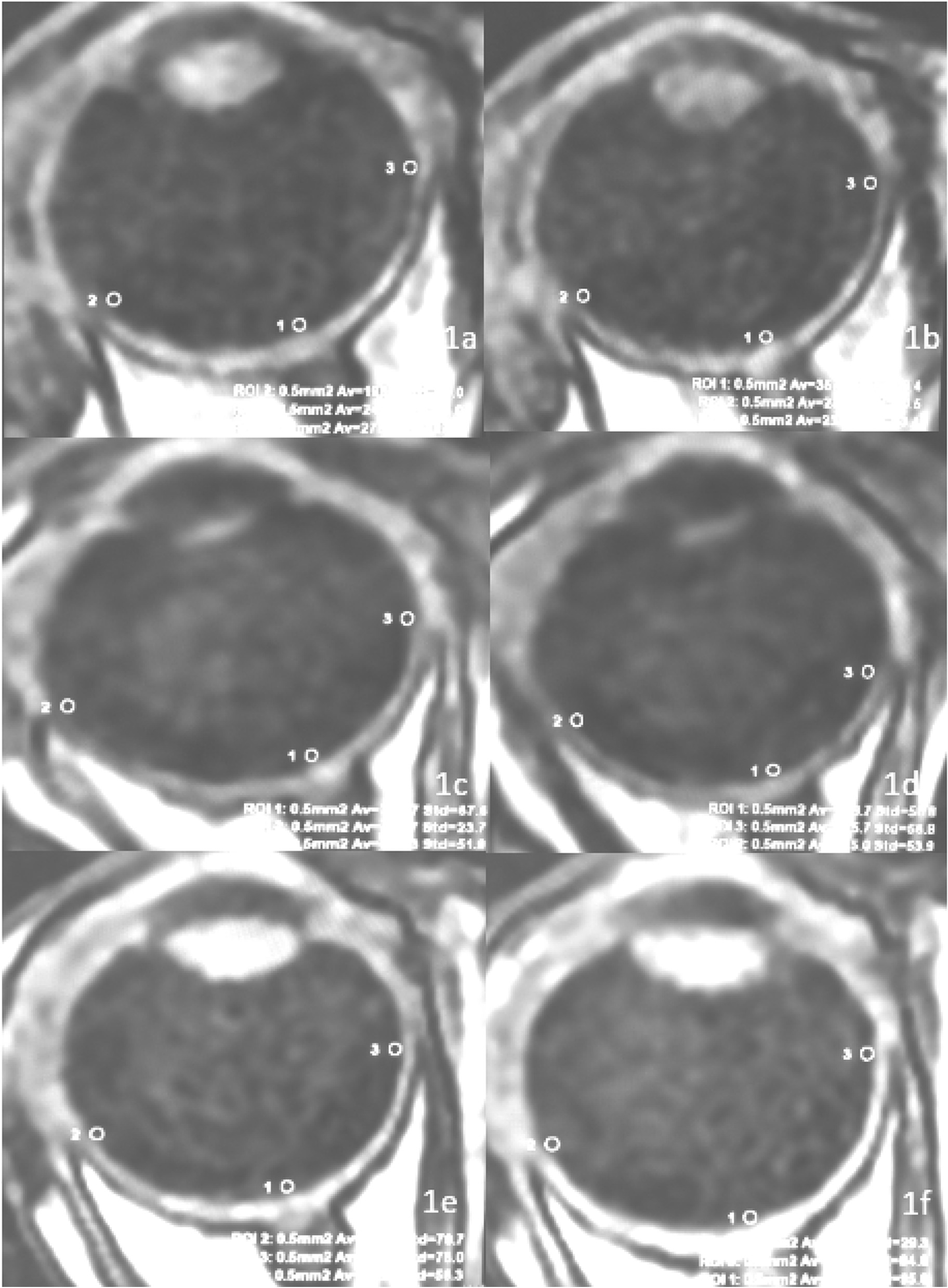
The region of interest ranges for the retinopathy group (**a**, **b**), the non-retinopathy group (**c**, **d**), and the control group (**e**, **f**)

Data analysis was performed using a GE Advantage Workstation 4.7 post-processing workstation and READY View software. Data were measured by two imaging physicians, each with 10 years of experience, and the corresponding values were averaged twice. The largest fault layer of the optic papilla and a circle with a size of 0.5 pixels were selected at the junction of the retina–choroid complex and the vitreous fluid. Measurements of the extension of the optic disc area of the anterior retina (the optic disc area) and the inner rectus eye-ring were taken, and this area along with the point (nasal side) and the attachment of the lateral rectus eye muscle (the temporal side) were the preretinal vitreous regions of interest (ROIs), and the signal intensity changes for each of these three ROI regions were measured. The average value was calculated (Fig. 1), along with the △PO_2_ and the maximum slope, according to the relevant formulae. For each pixel of the preretinal vitreous, the following formula was used to calculate the △PO_2_ [16]: the time–signal intensity change rate, i.e., E = (S_(t)_ − S_(0)_)/S_(0)_, where S_(t)_ is the signal intensity value of a specific pixel when the time after oxygen absorption is t, and S_(0)_ is the signal intensity value of a specific pixel in the same position in the indoor air in the atmospheric environment. [16, 17].

Damage to the blood–preretinal barrier was indirectly reflected by the maximum slope value of the time–signal intensity changes after each set of oxygen inhalation. The maximum slope calculation formula was as follows: Maximum Slope = (SI_peak_ − SI_pre_)/(SI_pre_ × T_peak_) [18], where SI_peak_ is the signal strength when the signal intensity value reaches its maximum after oxygen absorption, and SI_pre_ is the inhalation room in the atmospheric environment. The signal intensity value in the air, i.e., T_peak,_ is the time corresponding to the maximum signal strength value after oxygen absorption.

### Statistical analysis

The SPSS Statistics 22.0 (IBM, Chicago, USA) software program was used to conduct the statistical analysis. Continuous variables were expressed as mean ± standard deviation, and discontinuous variables were expressed as a percentage. A Kolmogorov–Smirnov test was carried out to assess whether the measurement data were consistent with the normal distribution. An independent sample t-test was used to analyze whether there was a statistical difference between the DR group and the NDR group. Analysis of variance and multiple comparisons were used to determine whether there were differences between the groups in the oxygen tension. A paired t-test was used to analyze whether the left and right-eye oxygen tension within the same group showed statistical significance. The intraclass correlation coefficient (ICC) was used to analyze the consistency of the two observers, with ICC > 0.75 indicating an excellent correlation, and *P* < 0.05 was considered statistically significant.

## Results

### General patient characteristics

A total of 15 patients with type 2 diabetes without hypertension were included in this study and divided into the DR group (*n* = 10 eyes) and the NDR group (*n* = 20 eyes). A healthy control group (14 eyes) was also recruited. The DR group comprised 5 patients with diabetes and non-proliferative or proliferative retinopathy (a total of 10 eyes); they included 2 males and 3 females, aged 50–73 years old, with an average age of 56.2 ± 8.5. The disease duration range was 10–20 years with an average of 16.8 ± 3.7 years. In the NDR group, there were 10 cases (a total of 20 eyes), including 4 males and 6 females, aged 54–69 years, with an average age of 61 ± 4.8. The disease duration was 1–25 years with an average of 8.4 ± 6.4 years. The healthy control group included 7 people (a total of 14 eyes); there were 2 males and 5 females, aged 55–74 years, with an average age of 62.1 ± 6.2. These patients had no diabetes, eye disease, or systemic diseases with eye complications, and they all underwent an ophthalmology review to confirm the absence of any signs of disease. No participants had a history of hypertension, metal implants, and no history of claustrophobia. In addition, there were no statistical differences in gender, age, blood pressure, or disease duration (as relevant) between the three groups (*P* > 0.05). There was also no significant difference in fasting blood glucose or glycosylated hemoglobin between the DR and NDR groups (*P* > 0.05) (see Table 1).

**Table 1:** The general characteristics

Index	DR group (*n* = 10)	NDR group (*n* = 20)	Control group (*n* = 14)	*P*
Sex(male/female)	4/6	8/12	4/10	0.424
Age(years)	56.2 ± 8.52	61 ± 4.80	62.14 ± 6.27	0.313
Disease Duration(years)	16.8 ± 3.7	8.4 ± 6.4	–	0.621
FPG(mmol/L)	9.79 ± 2.88	9.40 ± 4.45	4.69 ± 0.77	0.869
HbA1c(%)	9.4 ± 1.93	7.66 ± 1.75	5.54 ± 0.29	0.119
SBP(mmHg)	123.59 ± 5.95	122.45 ± 5.54	125.45 ± 4.45	0.536
DBP(mmHg)	84.53 ± 5.42	83.74 ± 4.89	89.42 ± 4.42	0.836

### Changes in intraretinal oxygenation response before and after inhaling medical oxygen

**Fig. 2:**
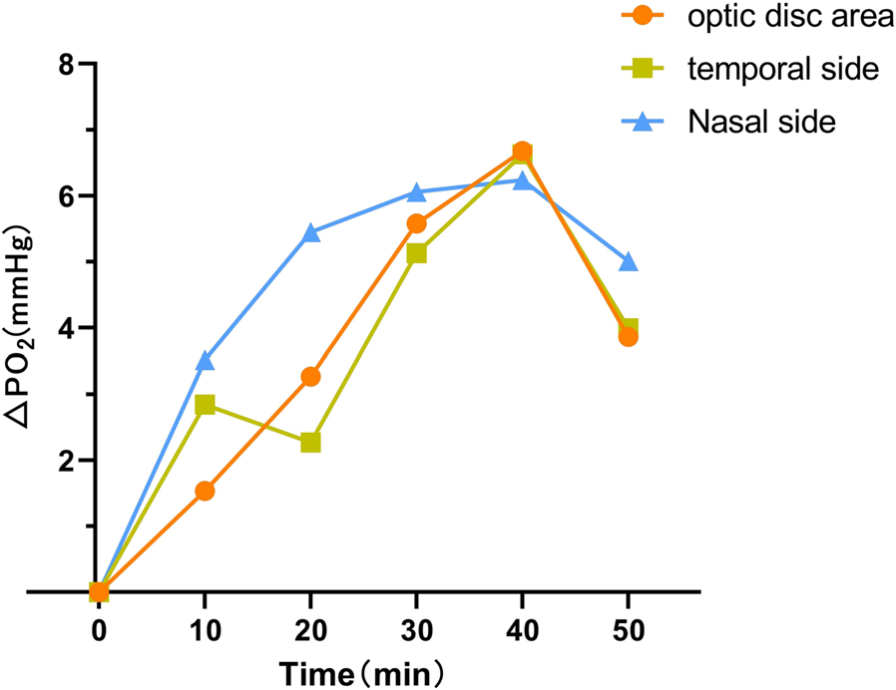
The time–oxygen partial pressure curves of the optic disc area, temporal side, and nasal side of the retinopathy group

**Fig. 3:**
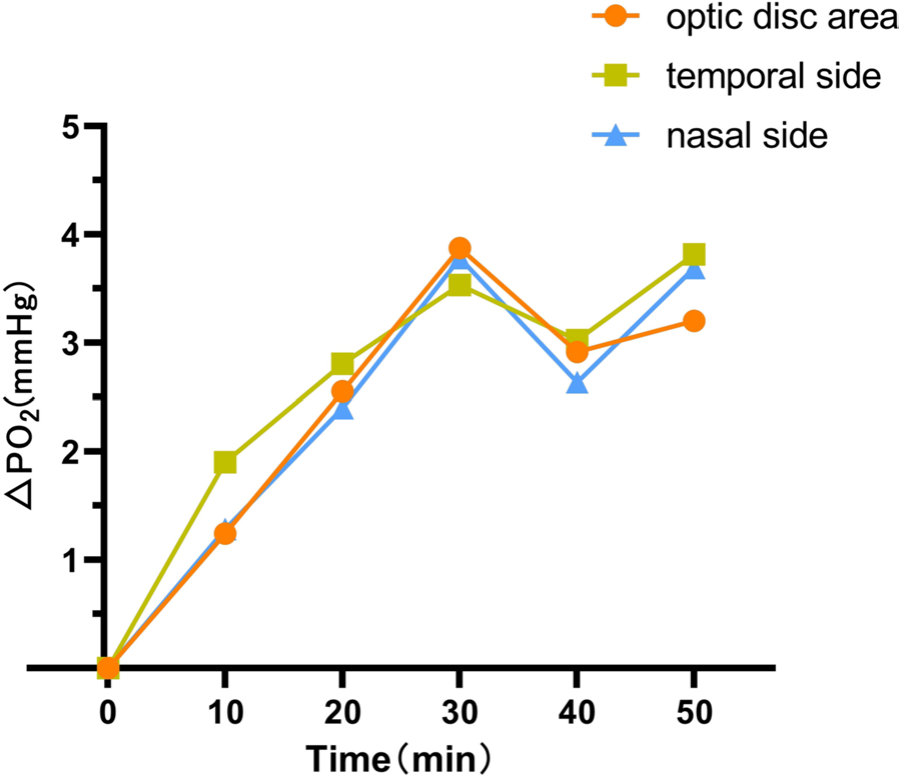
The time–oxygen partial pressure curves of the optic disc area, temporal side, and nasal side of the non-retinopathy group

**Fig. 4:**
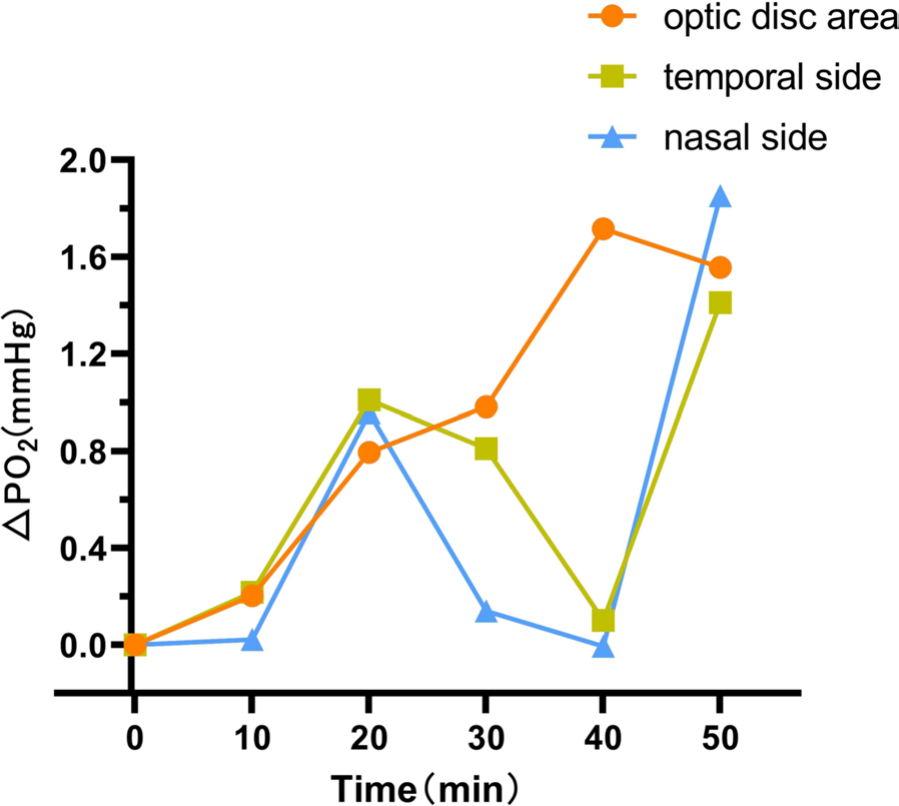
The time–oxygen partial pressure curves of the optic disc area, temporal side, and nasal side of the control group

In all three groups, the preretinal vitreous optic disc area, temporal side, and nasal side △PO_2_ levels increased with the increase in oxygen inhalation time (F = 100.19, *P* < 0.01), but the differences between the three groups were statistically significant (F = 7.54, *P* < 0.01). In the DR and NDR groups, the anterior vitreous △PO_2_ increased significantly at 10 min after oxygen inhalation, and the DR group peaked at 40 min after oxygen inhalation and then decreased rapidly. The NDR group peaked 30 min after oxygen inhalation and then showed a slow reduction. The control group showed a gradual increase with the increase in oxygen inhalation time and reached a peak 50 min after oxygen inhalation. The degree of oxygen tension was highest in the DR group, followed by the NDR group, with the control group showing the lowest value. However, there was no significant difference in oxygen tension at different sites in the three groups at the same time (*P* > 0.05) (see Tables 2 and 3 and Figs. 2, 3 and 4).

**Table 2:** Maximum slope analysis of the same part of the three groups

Index	DR group	NDR group	Control group	F value	*P* value
Video panel	1.10 ± 0.44	0.81 ± 0.40	0.45 ± 0.25	9.168	0.001
Temporal side	1.28 ± 0.63	1.05 ± 0.47	0.49 ± 0.39	8.813	0.001
Nasal side	1.73 ± 1.53	1.15 ± 0.77	0.81 ± 0.79	2.505	0.094

**Table 3:** Maximum slope multiple comparison (LSD) of the same part of the three groups

Index	DR group versus NDR group	NDR group versus control group	DR group versus control group
Video panel	0.054	0.008	0.000
Temporal side	0.245	0.002	0.000
Nasal side	0.139	0.335	0.031

### Analysis of the intraretinal oxygenation response of the anterior retinal vitreous at the same site

**Fig. 5:**
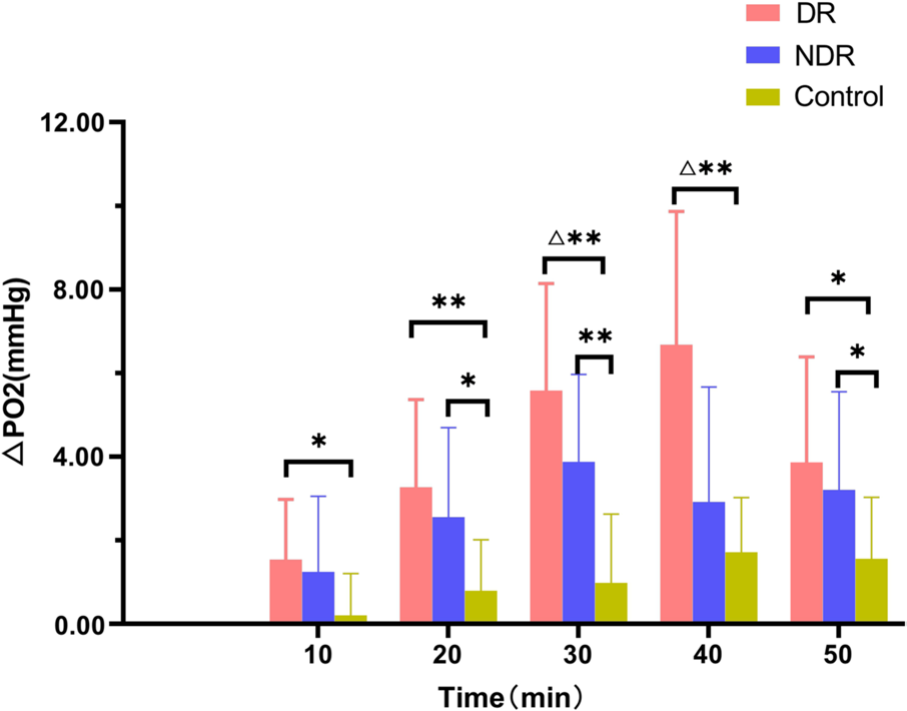
The oxygen tension values in the optic disc of the retinopathy, non-retinopathy, and control groups (△*P* < 0.05 vs. non-retinopathy group; *P < 0.05, statistically significant difference; **P < 0.01, statistically significant difference)

**Fig. 6:**
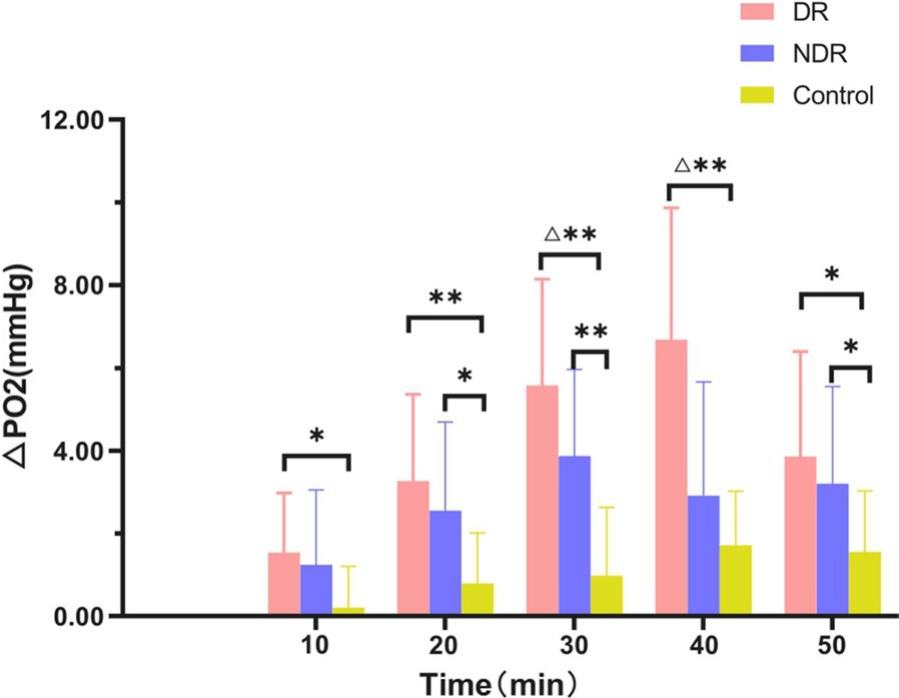
The temporal oxygen tension values in the retinopathy, non-retinopathy, and control groups (△*P* < 0.05 vs. non-retinopathy group; *P < 0.05, statistically significant difference; **P < 0.01, statistically significant difference)

**Fig. 7:**
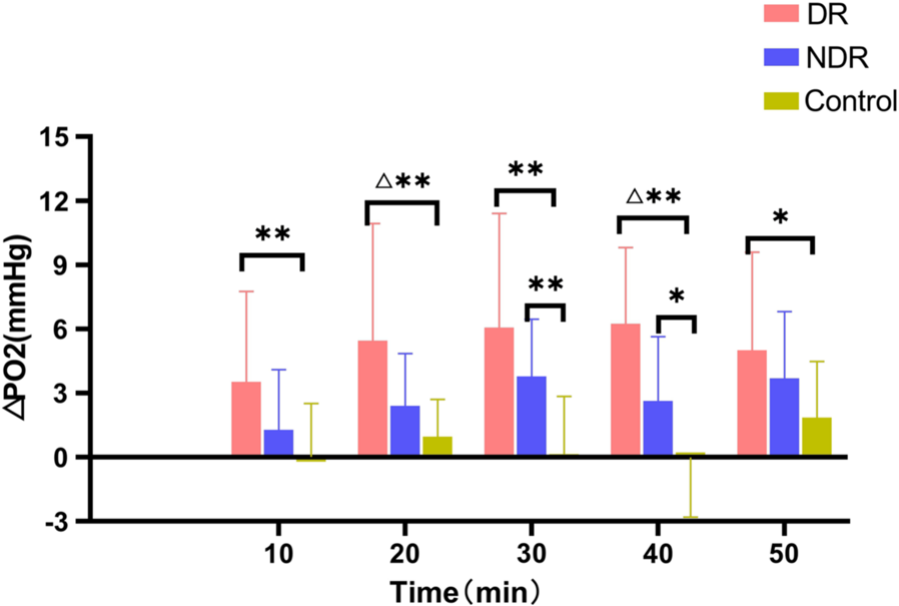
The nasal oxygen tension values in the retinopathy, non-retinopathy, and control groups (△*P* < 0.05 vs. non-retinopathy group; *P < 0.05, statistically significant difference; **P < 0.01, statistically significant difference)

There were significant differences in oxygen tension between the DR, NDR, and the control groups in the optic disc area from 20 to 50 min after oxygen inhalation (*P* < 0.05). There were also significant differences between the three groups in oxygen tension in the temporal side at 10, 20, 30, 40, and 50 min after oxygen inhalation (*P* < 0.05). Finally, there was a statistically significant difference in oxygen tension in the nasal side in all three groups between 10 and 40 min after oxygen inhalation (*P* < 0.05) (see Figs. 5, 6 and 7).

### Maximum slope comparison

There were statistically significant differences in the maximum slope between the three groups in the optic disc area and the temporal side but not the nasal side. There was a statistically significant difference between the DR and control groups in the increase of oxygen tension in the optic disc and the temporal and nasal sides. There were also statistically significant differences between the NDR and control groups in the optic disc area and the temporal side. However, the difference in the nasal side was not statistically significant. There were no significant differences in changes in oxygen tension between the optic disc and the temporal and nasal sides in the DR and NDR groups (see Table 4).

**Table 4:** Maximum slope analysis of the same part of the three groups

Group	Optic disc	Temporal side	Nasal side
Control group	0.45 ± 0.25	0.49 ± 0.39	0.81 ± 0.79
DR group	1.10 ± 0.44^a^	1.28 ± 0.63^a^	1.73 ± 1.53^a^
NDR group	0.81 ± 0.40^a^	1.05 ± 0.47^a^	1.15 ± 0.77

a: *P* < 0.05 when compared with control group; b: *P* < 0.05 when compared with DR group

## Discussion

In this study, 10 min after inhalation of medical oxygen, the △PO_2_ in the anterior retinal vitreous region of all three groups had increased and, with the prolongation of oxygen inhalation time, it showed an increasing trend. The increase in △PO_2_ was statistically significant. The different changes in △PO_2_ in the three groups were consistent with previous studies [10]. Compared with the control group, the optic disc results of the DR and NDR groups showed statistically significant differences in oxygen tension 20–50 min after oxygen inhalation (*P* < 0.05). With respect to the temporal site readings, the DR and NDR groups had marked differences in oxygen tension at 10, 30, 40, and 50 min after oxygen inhalation. The difference in oxygen tension at 40 min was statistically significant, suggesting that the exudation of oxygen molecules is related to the degree of vascular damage. It would appear that the microvessels of patients with diabetes are damaged by the high glucose state, and so oxygen uptake is affected. The different results of the NDR and the DR groups at the initial stage of oxygen inhalation further indicate that, with the progression of the disease, the vascular regulation function is gradually lost and vascular permeability and oxygen diffusion increase.

This study confirms the previous findings that elevated retinal oxygen tension increases the severity of diabetic retinopathy [19–21]. It is known that elevated oxygen tension indicates increased vascular permeability and that as the disease progresses, vascular regulation function is lost, and oxygen diffusion increases. Some researchers speculate that retinal circulation is regulated by metabolic demand, and blood flow increases during hypoxia, while diabetes-induced retinal nerve damage leads to a decrease in oxygen consumption, which leads to an increase in preretinal vitreous △PO_2_. In addition, since the retina in the ischemic state can cause the production of EGF, new blood vessels form in the anterior retinal region. However, the vascular tube walls are without smooth muscle components and are only composed of perforated endothelial cells and flaky basal membranes. As a result, the contractile capacity of the new capillaries is reduced, and oxygen molecules are dispersed to the preretinal vitreous, which increases the oxygen.

Oxygen molecules constitute a paramagnetic substance that can produce a change proportional to the change in oxygen concentration in the preretinal vitreous, and this change is characterized by a signal increase in the T1WI. The use of MRI is non-invasive and accurate, and measuring changes in the preretinal oxygen tension in the body [12, 22, 23], monitoring the different regions of the retina, and assessing the thickness of the retina can all contribute to the detection of early DR in patients with type 2 diabetes [24–26]. Furthermore, the detection of △PO_2_ using MRI can be as effective as taking oxygen probe electrode measurements [27].

In this study, the △PO_2_ levels in the two groups of patients with diabetes were found to be higher than in the healthy control group. The mechanism in this instance may be related to the fact that oxygen is cell-permeable and diffuses in the posterior vitreous. Therefore, △PO_2_ could be used as a monitoring index for judging DR [28]. The different manifestations of oxygen in the NDR and DR groups suggest that, as the disease progresses, the vascular regulatory function is lost and vascular permeability and oxygen diffusion increase.

There were significant differences in oxygen tension between the DR, NDR, and the control groups in the optic disc area from 20 to 50 min after oxygen inhalation (*P* < 0.05). The DR, NDR, and control group values in the temporal disc were similar at 10–30 min after oxygen inhalation. There were statistically significant differences in oxygen tension at 40–50 min. There were also statistically significant differences between the DR, NDR, and control groups in oxygen tension in the nasal side at 10–40 min after oxygen inhalation. These results are consistent with those in the existing literature [29].

The maximum slope of the DR group was significantly higher than that of the NDR group, suggesting that the exudation of oxygen molecules was related to the degree of vascular injury. The vascular lesions in the DR group were more severe than in the NDR group, and their ability to utilize oxygen molecules was decreased, causing the accumulation of oxygen molecules. The △PO_2_ values of the anterior vitreous of the optic disc, the temporal side, and the nasal side of the group increased over time, and the differences were statistically significant. However, the differences in △PO_2_ values among the optic disc, temporal, and nasal preretinal vitreous at the same time were not statistically significant. These results are consistent with the uniform dispersion of oxygen observed in the preretinal vitreous in previous studies.

Patients with hypertension as well as diabetes were not included in this study. Previous studies showed that hypertension aggravated the incidence of retinal exudates in patients with diabetes [30]. The △PO_2_ of the optic disc was higher than that of the temporal and nasal sides, and this was related to the increased expression of VEGF induced by hypertension. Elevated levels resulted in thickening of the basement membrane, increased vascular permeability, and neovascularization [15, 31]; in addition, elevated blood pressure also increased retinal blood flow.

In the current study, there was no significant difference in the oxygen tension between the optic disc area, nasal side, and temporal side in the same group, which was inconsistent with previous studies, but the effect of hypertension on the retina of patients with diabetes could not be considered here. Compared with the control group, the maximum slope of the optic disc area, temporal side, and nasal side of the DR group was statistically significant, which also indirectly suggested that △PO_2_ changes were related to the emergence of retinal neovascularization, and the incompleteness of the endothelial tissue itself determined that oxygen molecules were more likely to cross the blood vessel tissue.

The difference between the maximum slope of the three groups in this study was statistically significant compared with the difference between the optic disc area and the temporal side. On the other hand, the difference between the three groups on the nasal side was not statistically significant. This indicates that the optic disc area and temporal side were the sensitive areas showing lesions. The exact mechanism is not clear, and whether it is related to the optic disc area being the confluence of retinal arteries and veins as well as the site with the most neuromuscular blood vessels needs to be further explored.

In this study, the extension of the anterior vitreous optic disc (posterior optic disc), and the attachment points of the inner rectus eye-ring (nasal side) and the outer rectus eye-ring (temporal side) represented the ROIs of the anterior vitreous retina. This selection method minimized manual sampling errors, reduced retina–choroid complex interference with the analysis results, and ensured that the selection for each ROI was approximately the same.

This study had a number of limitations. It was a single-center trial, rather than being a randomized controlled trial, and the sample size was limited.

## Conclusion

The findings of this study indicate that 3D-SPGR echo MRI sequence technology is an effective method for detecting preretinal tension levels in patients with diabetes and that it can be used as one of the functional and imaging observation indicators for the early diagnosis of DR.

## Data Availability

We declared that materials described in the manuscript, including all relevant raw data, will be freely available to any scientist wishing to use them for non-commercial purposes, without breaching participant confidentiality. The datasets analysed during the current study available from the corresponding author on reasonable request.

## Abbreviations

DR: Diabetic retinopathy
NDR: Diabetic non-retinopathy
VEGF: Vascular endothelial growth factor
△PO_2_: Inner retinal oxygenation
3D-SPGR: Three-dimensional spoiled gradient recalled echo
TR: Repetition time
TE: Echo time
FOV: Field of view
NEX: Number of excitations
ROI: Region of interest
FA: Fluorescein angiography
FFA: Fundus fluorescein angiography
NPDR: Non-proliferative diabetic retinopathy
